# Patterns of respiratory tract infections in children under 5 years of age in a low–middle-income country

**DOI:** 10.1186/s42506-022-00118-0

**Published:** 2022-11-07

**Authors:** Nehal M. El-Koofy, Mortada H. El-Shabrawi, Basant A. Abd El-alim, Marwa M. Zein, Nora E. Badawi

**Affiliations:** 1 Department of Pediatrics, Faculty of Medicine, Cairo University, Cairo, Egypt; 2 Department of Public Health and Community Medicine, Faculty of Medicine, Cairo University, Cairo, Egypt

**Keywords:** Upper respiratory tract infections, Lower respiratory tract infections, Pneumonia, malnutrition, Crowding index, Children below 5 years, Breast feeding

## Abstract

**Background:**

Respiratory tract infections (RTIs) are among the most commonly encountered major public health problems, with a higher prevalence of lower RTIs among children and more generally the poor. The present study aimed to describe the pattern of respiratory tract infections in Egyptian children aged under 5 years and explore possible associations between socio-demographics and nutritional status and types of RTIs.

**Methods:**

Over 6 months beginning in September 2018 (including one winter season), a cross-sectional, observational, epidemiological study was conducted on a sample of patients with upper and lower RTIs diagnosed clinically and/or radiologically in the outpatient clinics at Cairo University Children’s Hospital in Egypt. An interview questionnaire was employed to collect socio-demographic and nutritional data. Heights/lengths and weights were measured and analyzed using the World Health Organization’s (WHO) Anthro Plus [Computer Program]. Patients with pneumonia (*n* = 28) were compared to 97 healthy children of the same age and sex.

**Results:**

The total number of children diagnosed with upper and lower respiratory infections was 611. Malnutrition was present in 12.4% of all children with upper and lower RTIs. Lower RTI and malnutrition were substantially more prevalent among children aged under 2 years (*p* = 0.048 and *p* < 0.001, respectively). The strongest predictor of lower RTI was a younger age (OR 0.797, CI 0.713–0.89, *p* < 0.001).

**Conclusion:**

At our center, approximately one-third of infections in under-fives were lower RTI. Malnutrition was one of the significant risk factors for lower RTI in children below 2 years. The nutritional status of infants and young children should be improved by encouraging exclusive breastfeeding during the first 6 months of life and strengthening the healthcare and nutritional counseling available for vulnerable children, particularly in rural regions.

## Introduction

Respiratory tract infections (RTIs) are among the most commonly encountered major public health problems [1], with a higher incidence of lower RTIs among children and more generally the poor [2]. Severe acute RTIs, particularly pneumonia in under-fives, lead to hospitalizations and mortality in 15% of cases [3, 4]. For practical purposes, pneumonia can be classified simply as pneumonia (accompanied by fast breathing but without chest in-drawing) or severe pneumonia (when accompanied by chest in-drawing and/or other danger signs) [4]. Low-income countries commonly experience a tenfold increase in mortality and a fiftyfold rise in the associated burden compared to developed countries [5]. Around 70% of childhood pneumonia hospitalization rates are among under-fives in the USA [6]. The World Health Organization (WHO) reported that in 2019, 740,180 children were died from pneumonia, which account for 14% of deaths in this age group [7]. According to UNICEF data; a child dies from pneumonia every 39 s [8].

In Egypt, the number of infants aged 1–11 months hospitalized for moderate or severe lower RTIs was 5135/100,000 for the period 2009–2012, with 40% of patients subsequently diagnosed with bronchiolitis [9]. A 2015 study on severe acute respiratory illness in Egyptian patients found that the causative organism was influenza A in almost half of cases, influenza B in 25% and respiratory syncytial virus in another 25%, with 35.3% of patients being children under 5 years of age [10].

Pneumonia can be caused by different bacterial, viral, or fungal agents, but only a third of children with bacterial pneumonia have access to life-saving antibiotics. Preventive strategies include adequate nutrition and healthy environmental conditions as well as immunization [7]. Understanding the risk factors for lower RTI enables us to predict and potentially avoid its emergence. However, the WHO determined that nearly all acute RTI-related deaths were caused by bacterial pneumonia, so instituting appropriate and timely antibacterial therapy is effective in reducing mortality from this illness [11].

Childhood wasting is the leading risk factor for mortality in under-fives with lower RTI worldwide, which may be attributed to insufficient breastfeeding practices and inadequate complementary feeding in early life [12]. Severe acute malnutrition, in particular, has been linked to higher death rates from pneumonia, diarrheal disease, and malaria, among other unfavorable socioeconomic circumstances, such as younger age, low birth weight, under-vaccination, parental smoking, early childhood respiratory damage due to indoor air pollution, other diseases, and overcrowding, which have also increased the prevalence and severity of these diseases [12, 13]. Malnutrition also has a detrimental effect on cognitive and physical development in children, perpetuating health inequalities [12]. The present study aims to describe the pattern of RTIs in under-fives who attended outpatient clinics at Cairo University Children’s Hospital, Egypt, explore possible associations between socio-demographic and nutritional status on types of RTIs, and identify possible predictors for lower RTIs.

## Methods

### Study design and setting

An epidemiologic–observational, cross-sectional study was conducted. Over 6 months beginning in September 2018, the study was conducted on patients visiting Aboelresh Children’s Hospital (a tertiary care hospital and one of Cairo University’s teaching hospitals).

### Study participants

The study was conducted with patients with upper RTIs—infections in the upper airways down to and including the larynx (e.g., rhinitis, sinusitis, otitis media, pharyngitis, follicular tonsillitis and croup) and lower RTIs (e.g., bronchitis, bronchiolitis, and pneumonia). Patients with at least one specific lower respiratory tract sign (e.g., tachypnea and chest wall indrawing), abnormal auscultatory findings (e.g., wheezes/crepitations or bronchial breath sounds), and an abnormal chest X-ray were deemed to have a lower RTI. Only patients who had consulted the practice for the first time in their current infection were included. Patients who were on corticosteroid therapy (i.e., asthmatics, unless they had symptoms and signs of an RTI) or had a persistent underlying medical condition, were excluded from the study.

### Sample size and type

The sample was a consecutive non-probability sample (patients meeting the selection criteria over 6 months; in one autumn and one winter season). A total of 611 children were diagnosed with upper (*n* = 308) and lower (*n* = 303) RTIs.

### Data collection tool and technique

An interview-guided questionnaire was employed to obtain socio-demographic data (e.g., age, sex, geographic residence (urban/rural), crowding index (more or less than two, where more than two individuals per bedroom was deemed overcrowded) [14], and the type of infant feeding). Heights (for children 24 months or older), recumbent lengths (for children under 24 months) and weights were measured and analyzed using the WHO’s Anthro Plus [Computer Program] [15]. The child’s age, weight, edema status (yes/no), length/height, and type of measurement (recumbent or standing) as well as their date of birth and visit date were inputted into the program. Measurements were converted to percentiles and *Z* scores. Children were described as normal or malnourished (wasted: a weight-for-length/height *z*-score <− 2 S.D.s or stunted: low height for age [16].

Children were divided into two age groups: those under two and those aged 2–5. Patients with pneumonia (*n* = 28) were compared to a control group of 97 age- and sex-matched normal healthy children who had no history of pneumonia past or present.

### Ethical considerations

The study was approved by the Kasr Al-Ainy School of Medicine’s Institutional Review Board, Cairo University (IRB number: I-161006, date of approval 25/9/2018), and patients were treated according to the Helsinki Declaration of biomedical ethics. After informing guardians about the study’s objectives, written informed consent was obtained from them.

### Statistical analysis

Data were revised for completeness and logical consistency. Pre-coded data were entered using Microsoft Office Excel Software Program 2016 and then transferred to the Statistical Package for Social Science version 25 for statistical analysis. Qualitative variables were described as frequencies and percentages. The chi-square and Fisher exact tests were performed, with a significance level of *p* < 0.05. A binary logistic regression model was used to determine the effect of independent factors (e.g., sex, age, feeding, crowding index, place of residence, and maternal and paternal education) on developing a lower RTI (dependent variable).

## Results

From late summer to autumn and winter, a total of 93,600 children aged up to 12 years presented to the outpatient department with various complaints, 60.4% of whom were under the age of 5. From the under-five patients attending the outpatient clinics during the study period, 611 had upper and lower RTIs (65.2/10000), of whom 303 had lower RTI (32.4/10000), 308 had upper RTI (32.9/10000), and 850 had other non-respiratory infections, including 158 cases of diarrheal disease.

By extrapolating the number of children in the representative sample to the Greater Cairo area, lower RTIs would be present in 22.1/100000 children under 5 years, 5.35/1000 patients aged under 5 years, and 35.64/100 patients attending the pediatrics outpatients with all types of infections. Diarrheal disease was the third-most common infection (Table 1). Other infections included those involving all systems other than the respiratory and gastrointestinal systems.

**Table 1:** Patient diagnoses, numbers, and rates of infection during the study period in Cairo University Children’s Hospital, September 2018–March 2019 (*n* = 611)

Lower RTI patients	303
Upper RTI patients	308
Patients with diarrheal disease	158
All types of other (non-respiratory) infections	850
Total patients attending outpatients	93600
Estimated population of Greater Cairo^a^	20485000
Estimated population of Greater Cairo< 5 years of age^b^	3072750
Proportion of lower RTI from 100 patients with infection attending outpatients	35.647059
Lower RTI patients per 1000 patients attending outpatients	3.2371795
Lower RTI patients per 1000 patients < 5 years attending outpatients	5.35
Rate of lower RTI among under-fives/100,000	9.86087

Indicators:

–Proportion of patients with a lower RTI to all those with infections attending outpatients = Lower RTI/patients with infection attending outpatient × 100

–Proportion of patients with a lower RTI to all those attending outpatients = Lower RTI/patients attending outpatients × 1000

–Rate of lower RTI in under-fives = Lower RTI/under-fives in a certain year and locality × 100,000

*RTI* respiratory tract infection

^a^Greater Cairo population 2019 https://populationstat.com/egypt/cairo

^b^Children under 5 years old in Egypt represent about 15% of the total population according to the population pyramid https://www.populationpyramid.net/egypt/2019/

The mean age of children with upper and lower RTIs (*n* = 611) was 2.3 ± 1.5 years, with a median crowding index of 2.3 (IQR 2–3). Children aged under 2 years represented 286 (46.8%) of the sample, while those aged 2–5 years comprised 325 (53.2%). The percentages of urban and rural residents were 68.7% and 31.3%, respectively. Malnutrition (underweight, wasting, or stunting) was detected in 76 (12.4%) of the study group, with 30 (4.9%) of them experiencing stunting.

There was no significant difference in nutritional status between children with upper and lower RTIs, where malnutrition occurred in 40 cases (13%) with upper RTI compared to 36 (11.9%) with lower RTI (*p* = 0.679). However, children under 2 years had a higher prevalence of lower RTI (*p* = 0.048). There were no differences in the sex ratio, paternal education, crowding index, feeding during the first 6 months of life, or geographic residence (Table 2). Of the study group, 409/611 (66.9 %) were breastfed exclusively, whereas 23% were formula-fed.

**Table 2:** Socio-demographic and nutritional differences associated with upper and lower RTI patients under 5 years of age, Cairo University Children’s Hospital September 2018–March 2019 (*n* = 611)

Socio-demographic and nutritional variables	Upper RTI		Lower RTI		*P* value
	(*n* = 308)		(*n* = 303)		
	Number	Percent	Number	Percent	
**Age group**					
< 2 years	132	46.2	154	53.8	0.048*
2–5 years	176	54.2	149	45.8	
**Sex**					
Male	165	50.9	159	49.1	0.786
Female	143	49.8	144	50.2	
**Residence**					
Urban	218	51.9	202	48.1	0.273
Rural	90	47.1	101	52.9	
**Crowding Index**					
> 2	150	48.9	157	51.1	0.441
≤ 2	158	52	146	48	
**Paternal education**					
Not educated	85	54.1	72	45.9	0.001*
Primary	46	47.4	51	52.6	
Secondary	94	41.6	132	58.4	
University or higher	83	63.4	48	36.6	
**Maternal education**					
Not educated	98	56.6	75	43.4	0.266
Primary	75	47.2	84	52.8	
Secondary	89	47.6	98	52.4	
University or higher	46	50	46	50	
**Feeding**					
Breastfeeding	198	48.4	211	51.6	0.371
Artificial feeding	77	54.6	64	45.4	
Mixed	33	54.1	28	45.9	
**Nutritional status**					
Normal	268	87	267	88.1	0.679
Malnourished	40	13	36	11.9	

*RTI* respiratory tract infection

*Significant

Table 3 compares children with normal nutritional status with those with malnutrition who have a lower RTI. Malnutrition was more likely to be found in children under 2 years of age than in those aged 2 to 5 (*p* < 0.001). Further, rural children with lower RTI were considerably more likely to be malnourished, whereas children with adequate nutritional status were more likely to live in urban areas (*p* = 0.008). There were no differences between urban and rural patients concerning paternal education, maternal education, or history of early infancy breastfeeding.

**Table 3:** Socio-demographic differences between normal and malnourished children under 5 years of age with upper and lower RTIs (combined) in the study group, Cairo University Children’s Hospital September 2018–March 2019 (*n* = 611)

	Malnourished (*n* = 76)		Normal (*n* = 535)		*P* value
	Number	Percent	Number	Percent	
**Sex**					
Male	43	56.6	281	52.5	0.507
Female	33	43.4	254	47.5	
**Age group**					
< 2 years	56	73.7	230	43	<0.001*
2–5 years	20	26.3	305	57	
**Residence**					
Urban	50	65.8	370	69.2	0.553
Rural	26	34.2	165	30.8	
**Crowding Index**					
> 2	36	47.4	271	50.7	0.592
≤ 2	40	52.6	264	49.3	
**Paternal education**					
None	18	23.7	139	26	0.873
Primary	12	15.8	85	15.9	
Secondary	27	35.5	199	37.2	
Tertiary	19	25	112	20.9	
**Maternal education**					
Not educated	22	28.9	151	28.2	0.939
Primary	19	25	140	26.2	
Secondary	25	32.9	162	30.3	
Tertiary	10	13.2	82	15.3	
**Feeding in the first 6 months of life**					
Breastfeeding	48	63.2	361	67.5	0.209
Artificial feeding	23	30.3	118	22.1	
Mixed feeding	5	6.6	56	10.5	

*Significant

Pneumonia was diagnosed in 28 children, representing 9.2% of the 303 patients with a lower RTI. They were compared to 97 normal healthy children with no history of lower RTI or significant or recurrent upper RTI. Parental education was significantly lower (*p* = 0.007) (Table 4).

**Table 4:** Comparison between pneumonia cases (*n* = 28) and controls (*n* = 97) regarding the socio-demographic factors in under-fives, Cairo University Children’s Hospital September 2018–March 2019

Socio-demographic and nutritional variables	Controls (***n*** = 97)		Pneumonia cases (***n*** = 28)		*P* value
	Number	Percent	Number	Percent	
**Sex**					
Male	46	48	15	53.6	0.684
Female	51	52	13	46.4	
**Residence**					
Urban	74	76	20	71.4	0.62
Rural	23	24	8	28.6	
**Age**					
> 2 years	46	47	15	53.6	0.539
2–5 years	51	53	13	46.4	
**Crowding index**					
> 2	46	47.4	15	53.6	0.455
≤ 2	51	52.6	13	46.4	
**Paternal education**					
Not educated	7	7.2	7	25	0.009*
Primary	7	7.2	1	3.6	
Secondary	39	40.2	15	53.6	
University or higher	44	45.4	5	17.9	
**Maternal education**					
Not educated	9	9.3	6	21.4	0.093
Primary	17	17.5	5	17.9	
Secondary	31	32	12	42.9	
University or higher	40	41.2	5	17.9	
**Nutritional status**					
Normal	97	100	27	96.4	0.68
Wasted	0	0	1	3.6	

*Significant

Direct logistic regression analysis was performed to assess the impact of some factors on the likelihood of children acquiring a lower RTI. The model included seven independent variables: sex, age in years, feeding, residence, crowding index, and maternal and paternal education. Among these variables, only age of less than 2 years is a significant predictor of lower RTI (*p* < 0.001). Every year unit increase in a child’s age made them 0.797 times less likely to develop lower RTI (Table 5).

**Table 5:** Predictors of lower RTIs in children under 5 years of age, Cairo University Children’s Hospital September 2018–March 2019

Variable	***B***	***P*** value	Odds ratio	95% CI for odds ratio	
				Lower	Upper
Sex (male/female)	− .082	.623	.921	.665	1.277
Age (years)	− .227	**< 0.001**	.797	.713	.890
Residence (urban/rural)	− .185	.302	.831	.585	1.181
Crowding index (≤, > 2)	.317	.180	1.373	.864	2.182
Paternal education (educated/not)	− .123	.557	.884	.587	1.333
Maternal education (educated/not)	− .266	.187	.766	.516	1.138
Early infant feeding (Breast milk/formula)	.277	.119	1.319	.931	1.869

## Discussion

Acute upper and lower RTIs were equally frequent in male and female children during our study period, with a lower RTI prevalence of 9.86/100,000 among under-fives. Male children were found to have a higher incidence of RTI in previous investigations in Bangladesh and Iraq [17, 18], whereas female children were shown to have a higher incidence in an Indian study [19]. Females exhibit more significant immune responses than males across all age groups, implying that the sex differences may be genetic. However, there are also sex-determined differences in response to prenatal nutrition, resulting in the epigenetic reprogramming of immune responses [18]. Breastfeeding benefits female infants more than males, since breastfed female newborns had a lower risk of neonatal RTI [19].

This study detected lower RTIs in approximately one-third of the children younger than 5 years presenting to our hospital with all types of infections. The greatest predictor of lower RTI was revealed in children aged less than 2 years (OR 0.797, CI 0.713–0.89, *p* < 0.001). Previous studies in Bangladesh and India indicated that rates of both lower and upper RTIs decreased as children aged beyond 2 years [17, 19]. Immune immaturity in the first 2 years of birth explains the increased prevalence of RTIs [17].

Moreover, children under 2 years had the highest incidence of malnutrition (*p* < 0.001). Infants and young children are most vulnerable to malnutrition due to their high nutritional requirements during this period of rapid growth. Undernutrition, which presents as underweight, wasting, stunting, and/or micronutrient deficiencies, is the leading cause of growth failure worldwide, with stunting found to affect 13% of Egyptian children under 5 years in the most recent surveys [20, 21].

In the present study, malnourished patients with lower RTI were more likely to live in rural areas than nourished patients with lower RTI (*p* = 0.008), indicating that lower socioeconomic status is a significant risk factor for both wasting and lower RTI. Rural life in Egypt has several characteristics that expose children to lower RTI, including inadequate health services and a combination of macro and micronutrient deficiencies among rural Egyptians [20].

Furthermore, many children in countries like Egypt, categorized as low–middle income, have restricted access to more expensive animal-based meals and suffer from hidden hunger, and a lack of micronutrients (vitamins and minerals) despite adequate calorie intake [22]. The Egyptian Demographic and Health Survey 2014 recorded increases in rates of underweight and wasting in children under 5 (occurring in 6% and 8% of children, up from 4% and 3%, respectively) and a slight decline in stunting (from 23 to 21%) since 2000 [21].

Exclusive breastfeeding is unrivaled throughout the first 6 months of life to improve immunity, health, growth, and development and should be continued until the child reaches the age of two. Children who are exclusively breastfed during the first 6 months of life in developing countries experience a 30–42% reduction in the incidence of acute RTIs compared to those who are not [23]. According to UNICEF 2017 data, breastfeeding rates in low-, middle-, and high-income countries were 97.6%, 95.6%, and 78.8%, respectively, with 95.7% of Egyptian children having been breastfed [24]. Only 66.4% of our study group with RTIs was exclusively breastfed, while 23% received only formula. These depressing statistics may reflect a recent shift away from breastfeeding. Interventions to prevent protein-energy malnutrition should prioritize promoting exclusive breastfeeding in the first 6 months of life by identifying and addressing barriers. It has been found that growth failure occurs most frequently between the ages of 3 and 18 months when early weaning, inadequate complementary feeding, and protein-poor diets are introduced [25]. Children are also most vulnerable to infections during this period. The interplay between malnutrition and infection is likely to result in a vicious cycle. It has been demonstrated that poverty is the main cause of malnutrition [12].

The degree and distribution of protein-energy malnutrition and micronutrient deficiencies in the population depend on various factors, including educational level. Although parental education was not a predictor of lower RTI in this study, fathers’ education was significantly higher in cases of upper RTI than in those with lower RTI (*p* = 0.001) and controls compared to pneumonia cases (*p* = 0.007). However, there was no difference between the nourished and malnourished groups in this respect. Results of other studies conflicted, with many reporting a lower likelihood of RTI in children with educated parents and professional mothers (and those with high level of education). Others suggested no effect of maternal education on acute RTIs in young children [26, 27]. Education is a commonly used indicator of socioeconomic status and can lead to health-promoting behaviors such as better dietary choices, compliance with hygienic measures and immunization schedules, and refraining from smoking [25]. In general, lower socioeconomic circumstances result in poorer health [28].

The crowding index was similar in upper and lower RTI patients in our study, with a median of 2.3, indicating overcrowding in both groups. Household overcrowding is an indicator of low socioeconomic status, and other studies have found a substantial association between overcrowding and acute RTIs [27].

Diarrheal disease was the third-most-often-encountered infection during this investigation. Pathogens that cause acute diarrheal disease are more prevalent in low-income countries than in high-income countries due to a lack of safe water and access to hygiene. Diarrheal disease is the second leading cause of mortality in young children after pneumonia. Diarrheal disease and lower RTI, which may even occur concurrently in young children under the age of 5, have been connected to the same predisposing factors of malnutrition and unsanitary conditions [29].

Numerous studies have examined the influence of poor air quality, which is prevalent in urban areas, on the occurrence of acute RTI in children, concluding that air pollution causes damage that results in susceptibility to airway infections [30]. Children in rural areas may also be exposed to indoor air pollution that results from burning wood or dung [31]. The incidence of pneumonia can be reduced by proper nutrition, adequate immunization, and improved environmental conditions. While eradicating poverty in the long term would reduce child malnutrition, some immediate efforts should be implemented to address malnutrition in children [32].

### Study limitations

We did not test the children for micronutrient deficiencies. In addition, since this is a single center, albeit the largest of its kind in Egypt, it is hard to generalize our findings to the whole population. Our results have looked at associations with RTIs in children but have not determined their causations.

## Conclusion

During the autumn and winter months in Egypt, around half of infections in under-fives presenting to our center with RTIs had lower RTIs. The younger age (under 2 years) is the main risk factor for lower RTIs and malnutrition. The nutritional status of infants and young children should be improved by encouraging exclusive breastfeeding during the first 6 months of life and concentrating healthcare and nutritional counseling on vulnerable children, particularly in rural regions.

## Data Availability

The datasets used and/or analyzed during the current study are available upon reasonable request from the corresponding author.

## Abbreviations

RTI: Respiratory tract infection
WHO: World Health Organization
